# Association of Saliva Fluoride Level and Socioeconomic Factors with Dental Caries in 3-6 Years Old Children in Tehran-Iran

**Published:** 2011

**Authors:** Massoud Amanlou, Shahin Jafari, Nazila Afzalianmand, Zinat Bahrampour Omrany, Hassan Farsam, Farzaneh Nabati, Kowsar Bagherzadeh

**Affiliations:** a *Department of Medicinal Chemistry, Faculty of Pharmacy and Pharmaceutical Sciences Research Centre, Tehran University of Medical Sciences, 14155-6451, Tehran, Iran.*; b *Department of Oral Medicine, Faculty of Dentistry, Tehran University of Medical Sciences, 14155-6451, Tehran, Iran.*

**Keywords:** Decayed missing filled teeth, Dental hygiene, Fluoride level, Ion specific electrode, Socioeconomic status

## Abstract

Previous studies have indicated that there may be a relationship between the salivary fluoride concentrations and dental caries while the emphasis was on dental caries in permanent teeth. The aim of this study was to assess the prevalence of dental caries and its predictors in 3-6 year-old children in Tehran, Iran. The other objective of this investigation was to clarify a relationship between salivary fluoride levels of the studied children and their socioeconomic situations. The study population consisted of 205 children aged 3-6 years living in Tehran. Each child was examined for dental caries (decayed missing filled teeth (DMFT)) and unstimulated whole mixed saliva was collected 2 h post-prandial. All of the saliva samples were analyzed for fluoride concentration using an ion-specific electrode. The children were then grouped according to their DMFT, salivary fluoride levels (ppm) and socioeconomic factors (parent’s education and occupation) that resulted in a statistically significant relationship. The children with (DMFT < 1) were shown to have a significantly higher salivary fluoride level (p < 0.001) than prone children caries (DMFT > 1). The obtained results indicated that the caries prevalence among 3-6 year-old children in Tehran – the capital of the Islamic republic of Iran – is as low compared with those, living in developed countries.

## Introduction

Dental health, a branch of general health, reflects the individual health habits and the general health behavior in various ways (1). Life-style and living dynamics affect the oral health habits and consequently dental health (1, 2). Various factors such as dietary intake (3), bacterial infections (4), salivary flow-rate and viscosity (5), tooth type and morphology (6), topical and systemic use of fluorides (7) and socioeconomic parameters (2, 8), affect the prevalence of dental caries both in primary and permanent teeth. 

The prevalence of dental caries in developing countries is lower than that of developed countries (8-13). But in certain developing countries, rapid urbanization and advancement of socioeconomic condition have been associated with a sharp increase in caries prevalence (8, 13). The extent of dental caries is assessed using decayed, missing and filled teeth index, either for deciduous dentition (decayed missing filled teeth (DMFT)) or for permanent dentition (DMFT) as described by WHO (13).

Fluoridation of drinking water is reported to be an effective factor in reducing dental caries especially in lower social classes (14). Other procedures of using fluorides such as topical, systemic, and dietary and *etc*. have also been presented to prevent dental carries (11, 12, 15). At the same time, there are conflicting reports on the association between fluoride usage and dental caries especially in Africa (12).

One of the goals of WHO for the oral health by the year 2000, was caries-free 50% of 5 to 6 year-old children (13). However, in a study on 1587 children aged 5 to 6 in China reported on 2001 (10), the caries prevalence was high and the mean DMFT of the urban and rural children were obtained to be 4.8 and 7.0, respectively (10). The DMFT index reported by WHO of 12 year-old children in Iran was reported to be 1.67 in 2000 (16). According to our literature survey, there are a few data on the dental caries status and saliva fluoride level (17-20). In addition, no reports were found to clarify a relation between basal saliva fluoride content and DMFT index in preschool children. 

The aim of this study was to determine the prevalence of the dental caries and its predictors in 3-6 year-old pre-school children in Tehran. Furthermore, a relationship was elucidated between salivary fluoride levels of these children and their socioeconomic situations.

## Experimental

The study was carried out at the department of oral medicine, Tehran University of Medical Sciences, Tehran, Iran. It was conducted in 5 randomly chosen 3 kindergartens affiliated to Welfare Organization in Tehran. A total of 230 children (aged 3-6 years) with good health whose parents were permanent residents of Tehran were randomly selected. Of these population 210 parents gave informed consent, 20 declined the study, and 5 children were excluded due to their orthodontic appliances. 

A questionnaire regarding dental hygiene and habits of child, parent’s education, occupation and socioeconomic status of the family was sent to parents for completion. The children were examined 2 h after lunch at room temperature (25 ± 1^°^C) within 2 days. On the first day, saliva samples were collected and on the second day, dental examinations were carried out by a trained dentist using the methods described by WHO (21). The children were seated in a class room. Their teeth were dried by cotton rolls, no radiographs were taken and examinations were performed with natural light. 

Caries status: decayed, missing and filled primary (DMFT) or permanent teeth (DMFT) and tooth surfaces were assessed and scored according to WHO criteria (21). 

*Apparatus*

A Metrohm 692 pH/Ion Meter with a Metrohm Fluoride ion selective electrode (6.0502.150) coupled with Metrohm reference electrode Ag/AgCl (6.0729.100), was used for fluoride measurements.

*Chemicals, reagents and materials*

CTDA (trans-1,2-diaminocyclohexane N,N,N΄,N΄-tetraacetic acid), NaCl, NaF, NaOH and glacial acetic acid were of analytical reagent (AR) grade and purchased from (E-Merck, Germany).

*Fluoride standards and aanalysis*

The analysis was performed using an ion-specific electrode and the method of standard additions. A series of eight fluoride standards were prepared using sodium fluoride in deionized water ranging from 0.020 ppm to 1.00 ppm. This range of fluoride ion concentration ensured the Ion Meter properly calibration for the quantitative determination of fluoride in the saliva samples. Total ionic strength adjusting buffer II (TISAB II) was prepared through mixing 4 g CDTA (trans-1,2-cyclohexanediaminetetraacetic acid), 57 mL of glacial acetic acid and 58 g NaCl in about 500 mL deionized water, adjusting to pH between 5-5.5 by adding 5 M NaOH (200 g L^-1^) and diluting to 1 L volume with deionized water according to Metrohm application bulletin NO.82/3 e (19). 

*Fluoride determination*

The collected saliva (2 mL) was transferred to a screw caped tube and kept in freezer at -20°C till the analysis time. The fluoride content was determined by the ion specific electrode after treating with TISAB II buffer (22, 23) using standard addition method. The fluoride content of Tehran drinking water was also determined employing the same procedure. The permission to carry out the study was granted by the tehran university of medical sciences research council as well as school authorities. The informed consent was obtained from the children’s parents and the study design was approved by the Ethics Committee of Tehran University of Medical Sciences.

*Data management*

Final calculation of the samples’ fluoride content applying the Ion Selective Electrode (ISE) meter readings (in mV) was carried out in Microsoft Excel 2003 using logarithmic regression. 

The obtained data was entered and analyzed using the SPSS software for PC version 11.0 (SPSS Inc. Chicago IL. USA), Pearson’s chi-squared test, Mantel-Haenzel test and Levent’s test. The significance level was set at 5%. The values were presented as Mean ± SD The chi-square tests were used for the proportions’ comparison. The Levent’s test was applied to check any possible significant differences between the DMFT score and the gender. The Mantel-Haenszel test was employed to check the significant differences between the socioeconomic factors distribution and the DMFT score. The logistic regression analysis was performed to determine the relationship between the factors related to DMFT. Multiple logistic regression analysis, using backward stepwise method, was applied to assess the magnitude of risk upon the socioeconomic factors as independent variables and with DMFT value as dependent variables. The independent variables were dichotomized as No = 0, Yes = 1, except age. The dependent variable (DMFT) was categorized as zero (DMFT < 1) = 0 and (DMFT > 1) = 1. 

## Results and Discussion

*Baseline data*

A total of 205 children (including 120 boys and 85 girls, between 3-6 years old) were entered this study. 104 children (50.7%) were caries-free (DMFT < 1) in their primary or permanent teeth. The number of the children with caries (DMFT > 1) was 101 (49.3%). The mean DMFT index for all children was 0.99 ± 0.13. The children gender did not show significant association with DMFT (for boys < 1, DMFT = 50%; for girls < 1, DMFT = 51.8%; p = 0.80). The mean age of children with DMFT < 1 and DMFT > 1 were 4.52 ± 0.98 years and 4.93 ± 0.92 years, respectively, that indicates the DMFT increases with the age (p < 0.1). 24% of the children brushed their teeth daily (either self-brushing or parental assisted brushing), 61% brushed time to time and 15% never brushed their teeth. A Significant difference (p < 0.05) was also observed between DMFT index and tooth brushing (Table 1). 

**Table 1:** Demographic and independent explanatory variables on DMFT among 205, 3-6 years old children

**Varianles**	**DMFT < 1** **(104)**	**DMFT > 1** **(101)**	**p- value**
**Gender**			0.80
** Female**	44	41	
** Male**	60	60	
**Age (years)**	4.52 ± 0.99	4.93 ± 0.92	0.1
**Fluoride concentration (ppm)**	2.04x10^-2^ ± 0.38x10^-2^	1.68x10^-2^ ± 0.37x10^-2^	0.001
**Father’s occupation**			0.05
** Unskilled worker**	4	6	
** Staff member**	57	45	
** Self-employde **	23	41	
** Employers/professional**	20	9	
**Father’s education**			0.001
***<*12 years**	1	15	
** = 12 years**	33	34	
** >12 years**	70	52	
**Mother’s occupation**			0.001
** House keeper**	19	42	
** Staff member**	85	59	
**Mother’s education**			0.001
** <12 years**	1	12	
** = 12 years**	35	47	
** >12 years**	68	42	
**Dental hygiene (tooth brushing)**			0.05
** Never**	9	22	
** Sometimes**	73	52	
** Always**	22	27	

The mean saliva fluoride level of children with DMFT < 1 was 2.04 x10^-2^ ± 0.38 x10^-2^ ppm and 1.68 x10^-2^ ± 0.37 x10^-2^ ppm for DMFT > 1. As it is seen in Table 1, the father’s occupation and education level showed significant differences (p < 0.03 and p < 0.001 respectively) with the children’s DMFT index. The same correlation was seen in DMFT index of the children, according to their mother’s occupation and education with p < 0.001 (Table 1).

Multiple logistic regression analysis showed that being a self-employed father is a risk factor for high dental caries after controlling for possible confounders (Table 2). In comparison with self-employed fathers, those children with staff member fathers were likely to have dental caries about 2.4 times more. The proportion of the children with caries experience (DMFT > 1), was significantly (p < 0.05) higher in those who did not use toothbrush (71%), compared to those who used toothbrush in bedtime occasionally (58.4%). This test also revealed that children, who did not use toothbrush, had statistically significant higher caries prevalence than the others. This analysis showed that the mother’s occupation and education level was not significantly associated with the dental carries prevalence of children. By increasing each unit of the saliva fluoride contents, the prevalence of the dental caries reduced up to 95%. 

**Table 2:** Stepwise multiple regression analysis between DMFT index and socioeconomic factors

**Variables **	**Β**	**SE (β)**	exp β*	**p- value**
**Fluoride concentration**	- 2.97	0.57	0.05	0.001
**Father’s occupation:**				
**Self-employed**	0.85	0.37	2.35	0.02
**Father’s education:**				
**< 12 years**	2.19	1.09	8.96	0.08
**Dental hygiene (tooth brushing)**	- 0.81	0.34	0.44	0.02

*: Estimated odd ratio

The results of this study (Table 1) show that 50.7% of the examined children were caries free, which is not so far from the goals of “a minimum of 50% by year 2000” and “90% by 2010” recommended and reported by WHO on oral and dental hygiene education programs for developing countries (13). In our study, from the various socio-economical factors which have been used to assess socioeconomic position (10, 24, 25), only the parents education and the employment status, the oral health status of children and the saliva fluoride concentration were put into consideration (Tables 1 and 2), since the other studies related to the child dental health status and the family socioeconomic position have shown that the adjustment of one social indicator for another, is impossible (26). In the univariate analysis, all variables except those related to gender and age of the children, were strongly associated with high dental caries. However, after adjusting each variable with other variables, only the saliva fluoride concentration, the fathers’ occupation and education level and the children oral/dental health status remained significant. As it is shown in Table 2, the education level is an important sign of socioeconomic position that is applied to both genders and the higher education level is generally predictive of better jobs, higher incomes, better housing and more reasonable dietetics (27, 28, 29). 

These findings show the vital role of socioeconomical situation of parents on the dental health status of their children. Parents with higher education and better income are more concerned about their children dental health. These findings are in consistent with previously reported data (30). However, this statement cannot be generalized since there are many other socio-cultural, genetical and environmental factors that can affect children’s dental health (3, 8, 29, 30).

The question that remains unclear, is why fathers’ education level was more important than those of mothers’, especially in developing countries (29). A univariable analysis of our data showed that the caries prevalence increases from 38.2% to 92.3% as the level of education decreases from higher educated mother into less educated ones. These results are consistent with those reported from south africa (31), namibia (32), saudi arabia (33) and other countries (34, 35) , while there are also conflicting reports for other developing countries (36, 37). Therefore, the other indicators of the socioeconomic status should be included in future studies of this population.

Poor dental health history and dental hygiene habits of parents have shown associations with children’s DMFT > 1. Mother is known to have a biological role in the first microbial colonization of her child’s teeth (38), but father seems to emerge as a significant contributor to child’s dental health when the mother is working out of house and the father has to contribute to taking care of children as reported for Finnish culture (39). According to the report for Finnish five year-old children (39), mothers’ caries histories and hygiene habits were better than those of fathers. Then it is logical that the fathers’ poor dental health is strongly associated with their children’s poor dental health. Perhaps in Iranian society, the role of the male as the head of the family is still very important despite the increase of women in the labor force or the fact that hygiene habits of the fathers were better than those of fathers in developed countries.

One of the objectives of this study was to determine the effect of basal saliva fluoride concentration on DMFT index. A significant difference (p < 0.001) was found between salivary fluoride content and DMFT index (Table 1) which reveals the effectiveness of salivary fluoride on children’s dental caries as reported by other investigators (17-20). The obtained results show that a very small variation in the salivary fluoride may reduce dental caries and raises in the intra-oral concentration of fluoride in the saliva on a long-term basis would be a valuable adjunct to the anti-caries armamentarium.

The city of Tehran has water fluoridation system, which benefits almost the whole population. However, the level of fluoride in drinking water is low (0.1-0.3 ppm), and as a result tooth brushing with fluoride toothpaste is the only reliable and constant source of fluoride for the present study sample. However, association between brushing teeth at least once a day with fluoride toothpaste in a fluoridated area of Tehran, and caries levels, suggests that the fluoride levels were insufficient to prevent dental caries. This observation suggests that in Tehran, there is room for further reduction of caries by targeting the provision of constant amount of fluoride in potable water. 

The results of this study show that caries is directly related to a low frequency of brushing in general and tooth brushing with fluorinated toothpaste appeared in specific to have strong impact on caries. In the present study, nearly 85% of the children used a toothbrush and the probability of having dental caries was almost twice as high in children who did not use a toothbrush compared to children who did use once daily. Our findings of the low prevalence of dental caries in this study are not in agreement with those of previous studies from different countries (40-45). In addition, the DMFT index found in this study (Table 1), was among the lowest in Asia and EMRO countries (16). For example, among 3-6 year-old children in this study, the prevalence rate in permanent dentition was 74.9% (DMFT 0.99), which was lower than reports from india (83%, DMFT 3.5), saudi arabia (73.5%, DMFT 5.54) and china (76.6%, 4.50), but higher than hong kong (DMFT 0.9) (16). 

The DMFT index for 3-6 year-old children in the present study was similar to the reports from most industrialized and developing countries such as australia (1.2), norway (1.4), the united kingdom (1.1), and the united states of america (1.1), but lower than that of previous study in iran (2.0) (16). 

The low caries prevalence seen in this study population, in comparison with previous reports from Iran (46, 47) and other countries may be partly since this study is not representative of the general population of 3-6 year-olds in tehran. Because of the realities of life in Iran, the general population surveys of 3-6 year-old children are difficult to conduct (46), and even a national survey of 12 year-old children was confined to the children who attended the school (21). 

Additionally, the sample comes from a relatively wealthy part of Iran. More studies, particularly in the poorer area of Iran are necessary to confirm this claim. Multicentre studies are needed to elucidate whether the same pattern occurs in other parts of iran with different socio-economical conditions, different patterns of food consumption, and different level of fluoride in tap water.

## Conclusion

This study was done to determine the prevalence of dental caries and its predictors in children aged 3 to 6 in tehran that resulted in low caries prevalence compared to similar studies. This study may provide information to the health educators, planners, and the other health professionals, who help reducing dental caries. The main goals of dental health programmers should be to achieve brushing-quality as a habit in children, to reduce the consumption of sweets, and to increase the knowledge on dental health. Further studies should be carried out to elucidate optimum fluoride intake by age groups and efficient procedure of delivery optimum fluoride as well as the socioeconomical predictors for a developing country compared to developed or industrialized countries. 
